# Cell models for Down syndrome-Alzheimer’s disease research

**DOI:** 10.1042/NS20210054

**Published:** 2022-04-08

**Authors:** Yixing Wu, Nicole R. West, Anita Bhattacharyya, Frances K. Wiseman

**Affiliations:** 1 U.K. Dementia Research Institute, Institute of Neurology, University College London, London, U.K.; 2 Cellular and Molecular Biology Graduate Program, University of Wisconsin-Madison, Madison, WI 53705, U.S.A.; 3 Waisman Center, University of Wisconsin-Madison, Madison, WI 53705, U.S.A.; 4 Department of Cell and Regenerative Biology, School of Medicine and Public Health, University of Wisconsin-Madison, Madison, WI 53705, U.S.A.; 5 LonDownS Consortium, London, U.K.

**Keywords:** Alzheimers disease, Down syndrome, induced pluripotent stem cells

## Abstract

Down syndrome (DS) is the most common chromosomal abnormality and leads to intellectual disability, increased risk of cardiac defects, and an altered immune response. Individuals with DS have an extra full or partial copy of chromosome 21 (trisomy 21) and are more likely to develop early-onset Alzheimer’s disease (AD) than the general population. Changes in expression of human chromosome 21 (Hsa21)-encoded genes, such as amyloid precursor protein (*APP*), play an important role in the pathogenesis of AD in DS (DS-AD). However, the mechanisms of DS-AD remain poorly understood. To date, several mouse models with an extra copy of genes syntenic to Hsa21 have been developed to characterise DS-AD-related phenotypes. Nonetheless, due to genetic and physiological differences between mouse and human, mouse models cannot faithfully recapitulate all features of DS-AD. Cells differentiated from human-induced pluripotent stem cells (iPSCs), isolated from individuals with genetic diseases, can be used to model disease-related cellular and molecular pathologies, including DS. In this review, we will discuss the limitations of mouse models of DS and how these can be addressed using recent advancements in modelling DS using human iPSCs and iPSC-mouse chimeras, and potential applications of iPSCs in preclinical studies for DS-AD.

## Introduction

### Overview on Down syndrome neurodevelopment

Trisomy of human chromosome 21 (Hsa21) was first discovered as the underlying cause of Down syndrome (DS, Ts21) in 1959 [1,2] and is the most common genetic cause of intellectual disability, affecting approximately 1 in 700 live births [3–5]. Hsa21, first sequenced in 2000, is the smallest human autosome and makes up ∼1–1.5% of the human genome [5]. Overexpression of Hsa21 genes and non-coding elements alters prenatal development of the brain, however, some effects do not appear until later in life [6–8]. Aberrant neurodevelopment in DS leads to overall smaller brain volumes and structural defects in cerebral cortex and cerebellum, affecting cognitive functions such as attention, learning, memory, and motor function to varying degrees [6,8–10]. A reduction in brain volume is detected as early as 15 gestational weeks in foetuses with DS, and by adulthood, brains of individuals with DS are ∼20% smaller than controls when corrected for their reduced body size [11,12]. While it is clear from studies of post-mortem tissue that this smaller volume is primarily due to a reduction in the number of neurons, we have a poor understanding of the causal underlying cellular deficits [13–22]. Further, the molecular mechanisms driving these anatomical abnormalities are largely unknown, which has resulted in potential treatments to enhance cognition in infants and children with DS that target symptoms rather than the basis of the disorder [10]. Importantly, it is not known whether or how these initial neurodevelopmental deficits may affect the progression of AD pathology in DS.

### Overview on Alzheimer’s disease

According to the World Health Organization (WHO), Alzheimer’s disease (AD) contributes to 60–70% of the dementia cases worldwide [23]. AD causes progressive loss of memory and reduction in cognitive function that leads to dementia and ultimately death [24]. Brain atrophy due to neural and synaptic loss is also detectable in AD patients [25]. Presence of the neuropathological hallmarks amyloid-β (Aβ) plaques and neurofibrillary tangles (NFTs), formed from misfolded microtubule-associated protein tau (MAPT), are necessary for disease diagnosis [26].

Although more than 90% of AD cases are late-onset (LOAD) and sporadic (sAD) with no known causal mutations [27], several disease-related mutations in the genes encoding, amyloid precursor protein (*APP*), presenilin 1 (*PSEN1*) and presenilin 2 (*PSEN2*) cause early-onset AD (EOAD). APP can be processed by amyloidogenic or non-amyloidogenic pathways. In the amyloidogenic pathway, APP is cleaved in a two-step process to form Aβ. PSEN1 and 2 are subunits of the γ-secretase complex that catalyses the second cleavage step of APP yielding Aβ [28]. Mutations in *PSEN1* and *PSEN2* cause an increase in Aβ production or result in a shift in the Aβ40/Aβ42 ratio favouring the formation of pathogenic aggregates [29], which drives AD development. Genetic association studies have identified several risk genes involved in multiple pathways for EOAD and LOAD [30], including most significantly the ε4 allele of the apolipoprotein E (*APOE*) [31,32] and the more recently identified chromosome 21-encoded gene, ADAM metallopeptidase with thrombospondin type 1 motif 1 (*ADAMTS1*) [33]. Despite the aetiology of AD not being fully understood, it is widely accepted that it is a complex disease that affects multiple cell types in the brain [34] and that immune response, endocytosis, lipid transport and vesicle trafficking modulate disease development [33,35].

### The association between AD and DS

**Figure 1:**
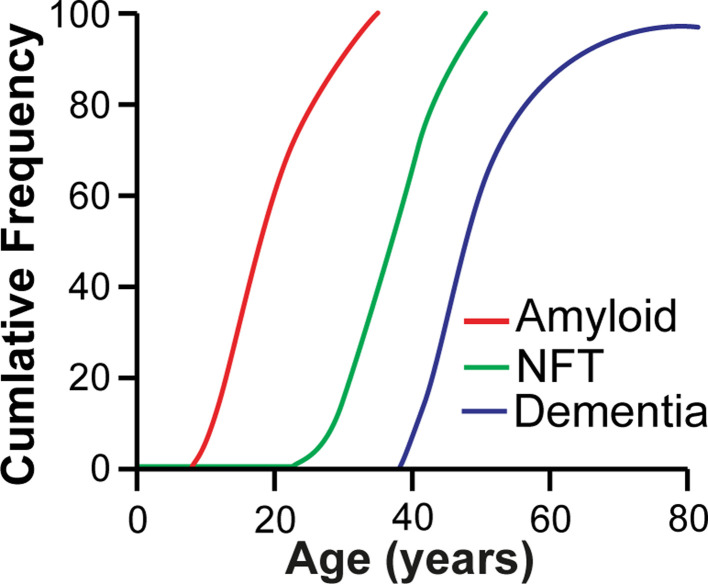
Schematic of the development of AD neuropathology and dementia in individuals who have DS People who have DS first develop Aβ deposition, NFTs and then go on to develop dementia in middle age.

People with DS have an extremely high risk of developing AD with extensive Aβ plaque accumulation occurring in most individuals by age 40 [36–38]. By the age of 60, approximately two-thirds of individuals with DS will have developed clinical dementia [39] (Figure 1). The pattern of cognitive decline is similar in individuals who have Alzheimer’s disease in Down syndrome (DS-AD) compared with AD, although occurring earlier in DS-AD [40], and individuals with DS-AD develop seizures more frequently than other forms of AD [41]. Triplication of a dosage-sensitive gene or genes on Hsa21 likely plays an important role in the pathogenesis of AD. *APP* is located on Hsa21 and duplication of *APP* in the absence of DS leads to EOAD [42,43]. Moreover, individuals with DS who do not have a third copy of *APP* do not develop AD neuropathology or dementia [44,45]. Thus, the additional copy of *APP* plays a central role in DS-AD. The pattern and type of Aβ accumulation in individuals with DS is similar to people with EOAD and LOAD, although occurrence of cerebral amyloid angiopathy is higher in DS-AD than EOAD and LOAD [46–50] (Figure 1). In recent years, whether other genes on Hsa21 also have roles in AD pathogenesis has been studied. Several Hsa21-encoded proteins are thought to be potential candidates for this altered biology, including dual-specificity tyrosine phosphorylation-regulated kinase 1A (DYRK1A) that phosphorylates tau [51,52] and APP [53], Synaptojanin 1 (SYNJ1) that is involved in endocytosis and membrane trafficking [54], β-Secretase 2 (BACE2) – a putative Aβ-degrading protease [55] and Cystatin B (CSTB), an endogenous inhibitor of cysteine cathepsins [56,57].

While chromosome 21 genes account for the majority of differentially expressed genes in DS, genes on other chromosomes are also differentially expressed and may also play a role in DS-AS progression [58]. Lockstone et al. found that *APOE*, while not an Hsa21 gene, is up-regulated in DS [58]. Recently, Bejanin et al. screened for the prevalence of the *APOE* ε4 AD-risk allele in 464 adults with DS [59]. They reported that 20.9% of individuals with DS had the *APOE* ε4 allele. These individuals had earlier cognitive decline and earlier clinical symptoms of AD compared with the 79.1% of DS individuals without the *APOE* ε4 allele [59], similar to findings in the general population and previous reports in individuals with DS [60–66]. Exploring the mechanistic roles of APOE isoforms and other non-Hsa21 genes in the pathogenesis of AD in DS is important for developing effective treatments for DS-AD.

## DS-AD mouse models and human tissue

### Uses and limitations of DS-AD mouse models

**Figure 2:**
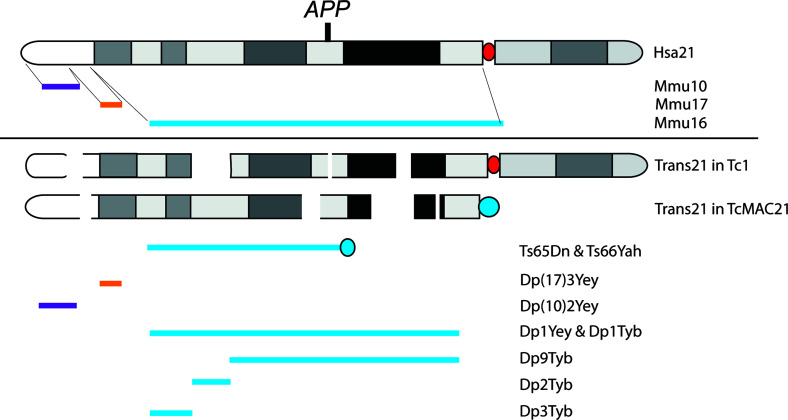
Schematic illustration of DS mouse models The regions of Mmu10 (purple), Mmu17 (orange) and Mmu16 (blue) that are homologous with Hsa21 (long arm) as indicated. The content of the transchromosome 21 in the Tc1 and TcMAC21 models with deletions and key rearrangements as indicated. The Tc1 mouse model has a human centromere (red circle). The TcMAC21, Ts65Dn and Ts66Yah have mouse centromeres (blue circle). The region Mmu16 with an additional copy in the Ts65Dn, Ts66Yah, Dp1Yey, Dp1Tyb, Dp2Tyb, Dp3Tyb and Dp9Tyb as indicated. The duplication of Mmu17 in the Dp(17)3Yey and the duplication of Mmu10 in Dp(10)2Yey as shown. The approximate human *APP* gene position is shown in bold, the TcMAC21, Ts65Dn, Ts66Yah, Dp1Yey, Dp1Tyb and Dp9Tyb models carry an additional copy of *APP/App*.

Mouse models overexpressing causal mutations of familial Alzheimer’s disease (fAD) are widely used in AD research, and recapitulate aspects of disease pathology [67], although differences in human and mouse biology limit the use of these systems for some key aspects of disease; most notably AD-neuroinflammation [68]. Moreover, compared with AD models, it is more challenging to generate DS mouse models because of the genetic complexity of the disorder and since orthologue genes of Hsa21 are located on regions of three mouse chromosomes (Mmu10, Mmu16 and Mmu17) [69]. However, to date, several DS mouse models have been developed [70,71] and have been used to study aspects of DS-AD (Figure 2).

One of the first mouse models of DS was the Ts65Dn [72] which has a partial extra copy of Mmu16 and is trisomic for approximately 55% of Hsa21 orthologous genes [73,74]. Ts65Dn mice exhibit learning impairment, locomotor hyperactivity, neurodegeneration and neuroinflammation [74,75], representing a number of the features of DS and AD. Using the Ts65Dn, Salehi et al. found that an increased level of *App* contributes to cholinergic neurodegeneration in the basal forebrain by disrupting NGF transport, providing insight into this feature of DS-AD [76]. Similarly, Garcia-Cerro et al. used the Ts65Dn to demonstrate the role of three copies of *Dyrk1A* in modulation of APP/Aβ biology [53] and Yin et al. used a pharmacological approach, targeting the kinase, to investigate changes of Tau biology in the model [77]. Moreover, use of an anti-Aβ vaccine in the Ts65Dn model alleviated some DS-AD-related phenotypes, demonstrating the importance of the peptide in disease mechanism [78]. The Ts65Dn model carries extra copies of some genes that are not orthologues of Hsa21 genes [73] and phenotypic drift has occurred in the mouse likely because of its complex genetic background limiting the utility of this model for future research [79].

More recently, a series of mouse models with extra copies of Mmu10, Mmu16 and Mmu17 genes, that are orthologous with Hsa21 have been generated including; Dp1Tyb, Dp2Tyb, Dp3Tyb and the Dp1Yey; Dp2Yey; Dp3Yey known as the DP16/10/17 ‘triple’ mouse model [80–83]. A recent study by Tosh et al. used segmental duplication mouse models (Dp2Tyb, Dp3Tyb, Dp2Yey and Dp3Yey) to understand which regions of Hsa21 can modulate Aβ aggregation [84]. The study identified that an extra copy of the genes located between *Mir802* and *Zbtb21* was sufficient to increase Aβ aggregation *in vivo*. However, these models lack some Hsa21 orthologues and cannot fully recapitulate trisomy of Hsa21 [84]. Moreover, Aβ plaques or aggregates do not form in the brains of models which carry an additional copy of the mouse *App* gene [75,81], likely because of differences in the biology of mouse and human APP/Aβ caused by key differences in the amino acid sequence between the species. Indeed, partial humanisation of mouse and rat *App* using knock-in approaches lead to a closer recapitulation of AD biology [85,86], and in the future such approaches may also lead to improved DS-AD rodent models.

The Tc1 ‘humanised’ transchromosomic mouse [formally called Tc(Hsa21)1TybEmcf], that carries an extra copy of approximately 75% of Hsa21 genes, was published in 2005 [87,88]. Tc1 mice show human DS-related defects in synaptic plasticity, cerebellar granule neurons and altered heart development [88]. Importantly, this model does not carry an extra copy of *APP* due to a rearrangement within the transchromosome [52,87], making Tc1 a useful tool for studying the role of other Hsa21 genes, independently of the triplication of *APP*, in the pathogenesis of AD. Using this approach Wiseman et al. demonstrated that Hsa21 genes other than *APP* increase Aβ deposition and exacerbate AD-related cognitive deficits [89]. However, during mouse development, random loss of the additional chromosome leads to mosaicism, limiting the ability to correlate genotype and phenotype in this system [88,90]. This model also lacks an additional copy of ∼25% of Hsa21 genes, such that it cannot be used to study the role of these missing genes in DS-AD [71,87].

Recently, a non-mosaic, transchromic DS mouse model, TcMAC21, was generated by cloning the long arm of Hsa21 as a mouse artificial chromosome [91]. TcMAC21 manifests DS-related features such as defects in memory, learning and synaptic plasticity, heart and craniofacial development as well as haematological abnormalities [91], making it by far the most genetically complete DS mouse model. Of note, TcMAC21 has elevated APP protein in the brain, but despite carrying an additional copy of human *APP*, Aβ plaques are not detected in the model [91], consistent with previous reports that humanisation of *App* is not sufficient to cause substantial Aβ accumulation in mice [92]. Further characterisation of this line and crossing it with mouse models of AD pathology will be needed to study plaque-associated DS-AD phenotypes.

Although DS mouse models have provided many insights into the causation and pathophysiology of both DS and AD, they are unable to fully reflect the human disorder because of the complex nature of genetic, transcriptional and translational regulation of human biology as well as the physiological and developmental differences between mouse and human [93–97]. In particular, comparative studies have indicated differences in neurotransmitter mechanisms between mouse and humans [98], and that some AD-specific patterns of gene expression are not recapitulated in the mouse [99], despite an overall good conservation of cell type. Moreover, differences between human and mouse astrocyte and microglia biology [99,100] may have particular implications for the modelling of neurodegenerative disease. Thus, although many aspects of DS and AD biology can be effectively modelled in mouse, additional research tools that capture key aspects of human biology that are not reproduced in rodents are also required to undertake research in these important areas.

### Uses and limitations of human tissue in DS-AD studies

Human tissue from individuals with DS and AD has long been an important source for immunohistochemical, biochemical and, more recently, transcriptomic analysis providing information about DS-AD-associated pathological changes. In the last decade, sequencing and genetics-based studies have elucidated the effects of full or partial copy of chromosome 21 (trisomy 21) on brain development [10,58,101,102], as well as AD-related pathology.

### Histology and biochemistry of AD-related phenotypes in DS

By studying post-mortem brain samples from individuals with DS across the lifespan, the pattern of Aβ plaque and NFTs formation has been determined to be broadly similar to that which occurs in AD, albeit commencing several decades earlier [103–107]. Aβ deposition is first seen in the parahippocampal gyrus in children with DS [36]. Loss of neurons in the entorhinal cortex occurs in both DS-AD and AD [108,109]. Coskun et al. show that mutations in mitochondrial DNA accumulate with age and are increased in DS-AD brains compared with age-matched controls [110] consistent with reports from AD in the general population [110,111]. Wilcock et al. analysed the expression of microglia markers in DS, DS-AD, and sAD tissue [112], revealing that elevated neuroinflammation occurs in the brains of people who have DS and unique neuroinflammatory phenotypes and microglia activation states occur in the DS-AD brain [112]. Additional studies have supported this seminal finding, showing differences in microglia morphology and cytokine profiles in the brains of people who have DS and DS-AD [113,114]. Notably altered cytokine changes predict cognitive decline DS-AD [115], consistent with reports of microglia activation correlating with increased tau across Braak stages in AD [116]. Further studies are needed to gain a better understanding of the contribution of different brain cell types to DS-AD pathology and cognitive decline.

### Transcriptomic studies to elucidate mechanism

The expression of genes throughout the genome is altered in the brain of people who have DS [58,101,102,117–121]. Gene expression profiling of foetus through adult post-mortem DS tissue has revealed that many, but not all, Hsa21 genes are up-regulated [58,101,102,117–121]. While triplication of *APP* is thought to be a main driver of DS-AD, Lockstone et al. found no evidence of increased APP abundance in the brain of adults who had DS [58]. In contrast, more recent studies have shown robust up-regulation of *APP* transcript and protein in the brains of individuals with DS and DS-AD [122,123]. The expression of other Hsa21 genes, including *DYRK1A*, *ADAMTS1*, *BACE2*, *RCAN1*, and non-Hsa21 genes of interest, including *APOE* and *NOTCH2*, is also increased in the brains of adults who have DS [58]. Using single-nucleus RNA-sequencing technology, Palmer et al. carried out a transcriptomics study in post-mortem prefrontal cortex from individuals with DS and euploid controls [123]. Consistent with recent histological and biochemical studies [113,114], this showed changes to microglia biology in both young and middle-aged adults who had DS and suggested a significant change in the ratio of inhibitory and excitatory neurons caused by trisomy of Hsa21 [123]. Further comparative single-nuclei RNA-sequencing studies of tissues from individuals who have DS and DS-AD (and equivalent tissues from the general population with and without AD) will provide critical new insights into how neurodevelopment and neurodegeneration are altered by trisomy of chromosome 21.

### Challenges and future approaches

Despite the significant information provided by studies of human post-mortem tissues, this research approach has a number of limitations. Although post-mortem tissue is typically matched by age, sex and post-mortem interval, it is not possible to account for all environmental differences that may affect phenotypes of interest. In addition, technical differences, such as fixation, method of processing the tissue or freezing the tissue, can affect results, making it difficult to compare findings from different studies and material sourced from different brain banks. Limited information on cellular processes can be obtained using post-mortem samples, and it is highly challenging to test molecular and cellular hypotheses as these provide information only at a static timepoint. Moreover, it is still challenging to obtain sufficient samples, both because of ethical constraints (such as ensuring appropriate informed consent from people who have an intellectual disability) and historical issues with accurate clinical diagnosis of dementia and mild cognitive impairment (MCI) in people with DS [124]. In particular, obtaining brain material from adults with DS that have not yet developed AD pathology is highly challenging because of the early development of pathology and can hamper adequate statistical power for many research questions. In 2013, The Academy of Medical Sciences released a report calling for increased collection of tissue at international biobanks [125]. Lawrence et al. surveyed U.K. researchers and determined their motivation for choice of tissue was availability of clinical data as well as sourcing from local tissue banks [124]. Further tissue banking from individuals who have DS or DS-AD who have undergone clinical phenotyping during their lifetime will help alleviate limitations of access to tissue.

## Cell models (non-pluripotent stem cells)

Cellular models can be used to address the limitations of animal preclinical models and human tissue studies, facilitating hypothesis-testing in a genetically and physiologically relevant system. Immortalised human cell lines and cells derived from affected individuals are commonly used to model and study cellular and molecular mechanisms in disorders and diseases, including DS and AD (Table 1). Human brain microvascular endothelial cells (hBMECs), human cerebral microvascular endothelial cells (hCMECs), human neuroblastoma cells (SHSY-5Y, SK-N-MC), human embryonic kidney cells (HEK293), human teratocarcinoma cells (NTera 2 or NT2/D1) and human lung cancer cells (CALU-3) are among the human cell lines used to screen potential therapeutics and have been valuable in understanding how overexpression of Hsa21 genes affects proliferation, differentiation, oxidative stress, Aβ accumulation, tau pathology and cell death in both DS and AD [126–141].

**Table 1:** Cellular Models used in DS and DS-AD research

Cell line/model	Source	Use	References
hBMECs	Human brain microvascular endothelial cells	Mimic the BBB	[134,135,38]
hCMECs	Human cerebral microvascular endothelial cells	Mimic the BBB	[136,137]
SHSY-5Y	Human neuroblastoma; subcloned from SK-N-MC cells	Neural-like	[127–129,132,137,139,140]
SK-N-MC	Human neuroblastoma	Neural-like	[138]
HEK293	Human embryonic kidney cell 293	Fundamental biological processes	[130,131]
NTera or NT2/D1	Human teratocarcinoma	Resemble neural precursor cells	[133]
CALU-3	Human lung adenocarcinoma	Mimic the nasal–brain barrier	[141]
Primary Cultures	Fibroblasts, astrocytes, neurons and neural stem/progenitor cells	Individual specific and disease relevant	[142–155]
hESCs	Human embryonic stem cells derived from blastocysts	Differentiate into cell types of interest; maintain genetic background of donor	[169–171]
iPSCs	Induced pluripotent stem cells reprogrammed from somatic cells	Differentiate into cell types of interest; maintain genetic background of donor	[55,180–231,234–242]
Organoids	iPSC-derived 3D model	3D culture; differentiate into cell types of interest; maintain genetic background of donor	[55,204,208,212,244,246–250]
Induced neurons (iNs)	iPSCs and somatic cells directly reprogrammed to neurons	Retain age-markers and genetic background of donor	[196,272–275]

A reduction in the GABA_A_ α3 subunit was detected in the hippocampus of DS foetal tissue [127]. To understand this feature, SH-SY5Y cells, which have neural origins, were treated with Aβ leading to a reduction in the GABA_A_ α3 subunit, suggesting that Aβ may play a role in regulating GABA_A_ receptor subunits [127]. Similarly, Krishtal et al. used SH-SY5Y cells to show that Aβ treatment caused neurite abnormalities, activated caspases, and caused cell death [128]. Moreover, increased *APP* expression in SH-SY5Y cells led to enhanced susceptibility to oxidative stress and cell death [129]. SH-SY5Y cells have also been used to investigate the role of vitamin A in neural differentiation because vitamin A deficiency is associated with AD and DS and induces neural differentiation by regulating mitochondrial morphology and function [139]. SH-SY5Y cells used to study *RCAN1* and oxidative stress revealed that inhibition of *RCAN1* reduces oxidative stress and apoptosis [140].

Non-neural HEK293 cells overexpressing *MAPT* formed pTau aggregates, which can be rescued by inhibition of kinase, glycogen synthase kinase 3 (GSK3), implicating GSK3 in the formation of pTau [130]. Notable changes in GSK3 activity have been reported in the Tc1 mouse models [52]. HEK293 cells overexpressing *DYRK1A* have hyperphosphorylated acetyl transferase, p300, and CREB-binding protein (CBP), revealing that *DYRK1A* may play a role in regulating enhancer activity and gene expression [131]. *DYRK1A* overexpression in SH-SY5Y cells reduced proliferation, and the sustained overexpression-induced cell cycle exit and premature neuronal differentiation, defects consistent with those seen in other trisomy 21 cellular models [132]. hBMECs and hCMECs are used to mimic the blood–brain barrier (BBB) and have been used as a model to study the BBB permeability to Aβ, BBB dysfunction and neuroinflammation, and to test uptake of potential AD therapeutics [134–138]. Quercetin, a potential AD therapy with low BBB permeability, was encased in liposomes with RMP-7 and lactoferrin. The liposome construct was permeable to the hBMEC BBB model, and Quercetin alleviated Aβ neurotoxicity in SK-N-MC cells [138]. Similar approaches may be used to understand how trisomy 21 impacts the BBB in DS-AD.

In summary, while these immortalised cells can easily be cultured and manipulated to study cellular defects that may be altered in DS and AD, these models do not carry trisomy 21 but only alter one or a few genes of interest, thus limiting them from fully recapitulating DS-AD biology.

### Cells derived from individuals with DS

Primary cell cultures of fibroblasts, neurons, astrocytes, and neural progenitor/stem cells derived from tissue of individuals who have DS, retain trisomy 21 and have revealed phenotypes associated with neurodegeneration, cell stress, and AD development.

Proteomics and transcriptomics of trisomy 21 primary fibroblasts have shown that Hsa21-encoded mRNAs and proteins are increased an average of approximately 1.5-fold and expression of other non-Hsa21 gene products is also altered, thus modelling a key aspect of DS biology [142]. Aneuploidy-associated stress response in cells leads to impaired cell proliferation, mitochondrial dysfunction, increased ROS, disrupted protein homoeostasis, trafficking deficits, accumulation of protein aggregates, and premature senescence in these cells, thus providing a system in which this key DS-AD relevant biology can be understood and potential treatments investigated [142–149].

Primary neurons and astrocytes can be derived from post-mortem foetal brain tissue and those from DS show increased ROS and undergo apoptosis compared with control cells [150] as well as dysfunctional mitochondria and altered processing of APP, leading to accumulation of insoluble Aβ [151]. With the capability to be differentiated into specific neural subtypes and glial cells, foetal tissue-derived neural stem cells (NSCs) can be used to study developmentally relevant disease mechanisms and pathology, which may overlap with neurodegenerative mechanisms. For example, altered synaptic pruning pathways impact both development and neuron degeneration in AD and DS-AD [152]. Trisomy 21 cultures reveal aberrant development of DS neurons, which may play a role in susceptibility to AD pathology later in life [14,153–155].

## Pluripotent stem cell models of DS-AD

**Figure 3:**
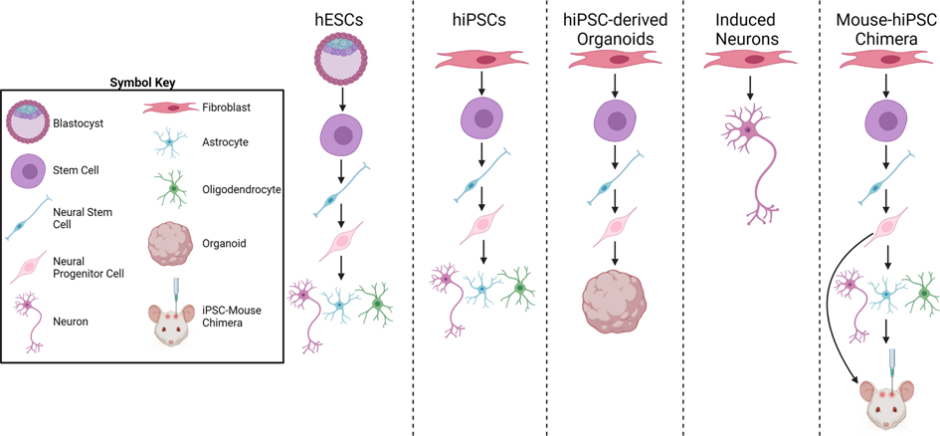
Schematic illustration of DS-AD cell models Patient-derived hESCs or hiPSCs are first patterned toward NSCs. They are then differentiated into neural progenitor cells and further differentiated into different cell types (astrocytes, neurons, and oligodendrocytes). Induced neurons skip progenitor stages by directly reprograming somatic cells into neurons. These new techniques and models are enhancing the research of DS-AD and have the potential for developing efficient treatments. Created with BioRender.com.

With the ability to be differentiated into many disease-relevant cells, human pluripotent stem cells (PSCs) are unmatched in their ability to model diseases and can also be used as a source of human cells for testing of therapeutics [156–167] (Figure 3). Human embryonic stem cells (hESCs) were successfully derived and cultured from human blastocysts in 1998 [168]. hESCs have since been derived from early embryos with aneuploidies, including trisomy 21 [169–171] and have developmental defects, including a reduction in pluripotency regulators leading to premature neuronal differentiation and increased cell death, consistent with mechanisms shown in other trisomy cell models as well as phenotypes seen in individuals with DS [169,170,172].

The use of hESCs in research is ethically controversial since they are derived from an early-stage human embryo [173–175]. Further, access to embryos with trisomy 21 is difficult, such that only limited DS and DS-AD research has been undertaken using hESCs. As an alternative, human somatic cells can be reprogrammed by introducing specific transcription factors (Oct3/4, Sox2, c-Myc, and Klf4; or, Oct3/4, Sox2, Nanog, and Lin28) that return the somatic cells to an undifferentiated, hESC-like state [176–179]. These induced pluripotent stem cells (iPSCs) have become an invaluable resource in research to model AD, DS, and DS-AD [180–185].

In 2011, iPSCs were first derived from individuals with autosomal-dominant, early-onset fAD caused by mutations in *PSEN1* and *PSEN2* [186] and subsequently from fAD individuals with a duplication of *APP* and individuals with sAD [187]. Neurons differentiated from these iPSCs recapitulate AD pathogenic features such as accumulation of Aβ [188,189] and increased pTau and GSK-3β validating these cells as an AD model [186,187]. For example, basal forebrain cholinergic neurons (BFCNs) are prone to degeneration in both DS and AD and have been differentiated from AD iPSCs to identify underlying cellular and molecular mechanisms of their vulnerability [190–192]. AD iPSCs have been used to understand the roles of AD-risk genes and the underlying mechanisms contributing to the onset and progression of the disease [193–201].

While these models have contributed significant knowledge of the pathophysiological mechanisms of the disease, a major limitation with 2D models is the inability to recapitulate all aspects of disease pathogenesis. Notably, these *in vitro* systems do not facilitate the development of extracellular Aβ plaques. Moreover, they do not fully replicate all of the age-dependent pathological features, and they also lack the complex interaction of multiple cell types, which are suggested to have a major role in AD development [202]. While AD iPSCs have been used extensively to elucidate underlying mechanisms of the disease, Israel et al. found iPSC lines generated from individuals with sAD and fAD with an *APP* duplication did not all display the same phenotypes [187]. Similarly, Kondo et al. found that seven AD iPSC lines did not recapitulate the same phenotypes [189], illustrating the underlying variability in this model system likely because of genetic differences between individuals.

iPSCs were first derived from cells from two individuals with DS in 2008 and retained trisomy 21, validating iPSC technology as a tool to study DS [203]. Subsequent studies generated trisomy iPSCs from both banked cells and directly from donor samples [55,185,203–231]. In early iPSC studies, disorder-specific cells were typically compared with an age- and sex-matched control. Inherent genetic human variation between controls and disorder made it hard to distinguish differences caused by the disorder from underlying genetic differences between individuals. The generation of isogenic pairs of trisomy and euploid iPSCs from mosaic trisomy 21 cells addressed this limitation [218,226]. However, mosaicism is rare and occurs in 2–4% of individuals with DS [232,233], limiting the generation of isogenic iPSC pairs by this approach. Another strategy to generate DS and control lines with limited genetic variability is to derive iPSCs from monozygotic twins discordant for DS [220,234]. Silencing of one copy of chromosome 21 in trisomy 21 iPSCs can also be accomplished [221,229]. One strategy is to co-opt function of *XIST*, the X-inactivation gene, in the *DYRK1A* locus on chromosome 21, allowing the *XIST* non-coding RNA to coat the chromosome and silence it [221]. When one copy of chromosome 21 was silenced in trisomy 21 iPSCs, proliferation and neural rosette formation defects were rescued [221]. These strategies provide models to study gene expression changes without confounds of genetic and epigenetic background.

Although iPSCs can be differentiated into various cell types, much of the trisomy 21 iPSC research has generated cells of the nervous system to investigate underlying mechanisms of intellectual disability. Trisomy 21 iPSC-derived neural progenitor cells (NPCs) and neurons have revealed deficits in cellular and molecular processes of neural development and maturation, as a result of extra copies of Hsa21 genes. Trisomy 21 NPCs have deficits in proliferation, differentiation, and migration [204,205,220–223,229]. Trisomy 21 neurons differentiated from NPCs have fewer processes, a reduced area, increased vulnerability to oxidative stress, and synaptic defects [213,224–226]. Furthermore, trisomy 21 NPCs differentiated into fewer neurons but more astrocytes and oligodendrocytes compared with controls, suggesting deficits in neurogenesis and a shift in the timing of the neuron–glial switch [154,219]. Compared with isogenic controls, trisomy 21 cells have decreased numbers of synapses, exhibit slower proliferation of neural progenitors, develop more double-stranded DNA breaks, and have increased Aβ levels, number of mitochondria, and markers of oxidative stress [218,226]. Transcriptomic analysis of iPSC-derived cells reveals that an additional copy of Hsa21 causes the differential expression of genes throughout the genome. Pathway analysis indicates changes in embryonic development, organ development, nervous system development, and cell adhesion along with reduced proliferation and increased apoptosis modelled in this system [220,226,229,234,235].

Trisomy 21 iPSC models have also been used to study the early pathogenic phenotypes associated with AD [55,217,218,224,236–240]. Trisomy 21 iPSC-derived neurons and hESC-derived neurons, develop AD pathology including Aβ and pTau accumulation [187,189,224,241,242]. Trisomy 21 iPSC-derived cortical neurons have increased insoluble Aβ, accumulate amyloid deposits [217,224], have increased hyperphosphorylated tau, and show that tau dissociates from axonal microtubules and relocalises to the cell body and dendrites, which are key pathological hallmarks of AD [217,224]. Ovchinnikov et al. used CRISPR methodology to delete the additional copy of *APP* in Trisomy 21 iPSCs and to up-regulate *APP* in euploid cells, showing the additional copy of *APP* is responsible for increased Aβ and the altered Aβ42/40 ratio that occurs in this model but is not responsible for tau-related phenotypes or increased apoptosis [213]. While iPSCs have been valuable in understanding DS and AD, neurons differentiated from iPSCs are functionally immature and do not retain age markers, limiting their use as a model for age-related aspects of AD [243].

### Three-dimensional cell cultures

While monolayer cultures provide insight into disease onset, progression, and drug discovery, they fail to recapitulate the dimensionality and complex circuitry of the brain. Three-dimensional organoid cultures derived from PSCs better model the brain *in vitro* and have been used to model AD phenotypes. With the overexpression of *APP* or *PSEN1* with fAD mutations, organoids accumulate Aβ plaques and aggregates of phosphorylated tau along with revealing that GSK3 regulates Aβ-mediated tau phosphorylation [244]. 3D organoid cultures of neurons respond to the addition of exogenous Aβ whereas 2D neuron cultures do not [245]. Kim et al. report Aβ aggregation after 6 weeks of differentiation and tau pathology after 10–14 weeks using organoids that overexpress *APP* or *PSEN1* with fAD mutations [246]. Using fAD patient-derived iPSCs with an *APP* duplication or mutation in *PSEN1*, Raja et al. found Aβ aggregation, hyperphosphorylated tau, and endosome abnormalities occur in an age-dependent manner in self-organising organoids [247]. To elucidate effects of glial cell types, Park et al. used a 3D triculture of AD-derived neurons and astrocytes with adult microglia in which Aβ and pTau accumulate and there is neuroinflammatory activity [248]. Thus, these 3D models exhibit features of AD that 2D cultures cannot.

Cerebral organoids generated from trisomy 21 iPSCs are smaller in size with decreased proliferation and fewer cortical neurons [55,204]. The DSCAM/PAK1 pathway, which regulates proliferation and is more active in DS, can be regulated with CRISPR interference (CRISPRi) and help normalise the size of the organoids [204]. Epigenetic ageing measured by Horvath clock DNA methylation is accelerated in DS organoids [249], concordant with the accelerated ageing hallmarks observed in DS tissue [250]. Recent work from Xu et al., indicated that the Hsa21-encoded *OLIG2* transcription factor causes an overproduction of progenitor cells and GABAergic interneurons [208]. Organoids will likely be more prevalent for assessing neurodevelopmental defects in DS in the future.

Recently, DS organoids have been used to study DS-AD. Organoids generated from iPSCs with fAD mutations or trisomy 21 accumulate structures similar to Aβ plaques and NFTs [212]. Similarly, Alić et al. reported Aβ deposits, hyperphosphorylated tau, and premature neuron loss in organoids derived from trisomy 21 iPSCs [55]. 3D organoids provide a better structural model of the brain and result in more mature cells, potentially making them a better model for DS-AD.

### Induced neurons

A key limitation of iPSC-derived cells is that they are developmentally immature, presenting a challenge to reflect age-dependent pathological features when modelling age-related diseases, such as AD. To better model age-related diseases, induced neurons (iNs) are directly reprogrammed into neurons from an affected individual’s somatic cells or iPSCs, skipping the NPCs stage [251,252]. Different neuron subtypes, including dopaminergic, motor, excitatory, inhibitory, serotonergic, cholinergic, and peripheral sensory neurons [236,251,253–265] induced by overexpressing specific combinations of transcription factors can currently be generated [266]. iNs that are converted directly from somatic cells maintain the individual’s epigenetic background at the time of cell collection, making them a valuable model for studying age-related neurodegeneration [267–271]. Mertens et al. report that AD iNs retain age markers of the donor individual, have a down-regulation of mature neuronal markers, and have up-regulation of immature neuron and progenitor-like pathways [196]. AD iPSC-derived neurons had no significant disease-related transcriptome signatures [196], corroborating earlier findings that excitatory iNs retain age-related signatures compared with iPSC-derived neurons from the same individuals [272]. Wang et al. used iNs for high-throughput screening to identify potential a drug candidate for AD that would lower tau [273]. Trisomy 21 iNs have the characteristic overexpression of Hsa21 genes at both the RNA and protein level, along with increased Aβ and pTau, increased synaptic vesicle release, and dysregulation of axonal transport [274]. Trisomy 21 iNs also show aneuploidy-associated stress response, dysregulated protein homoeostasis, up-regulation of the endoplasmic reticulum stress pathway, and increased cell death [275]. Treatment of iNs with 4-phenylbutyrate decreased protein aggregates and reduced cell apoptosis in the Ts21 iNs, suggesting that the aneuploidy stress may be a target for neurodegeneration in DS and DS-AD [275]. As a relatively new model, iNs have thus far yielded limited data on disease onset and progression in AD and DS-AD. Moreover, currently isogenic controls for Trisomy 21 iNs are lacking, and further refinement of this technology will ensure its utility to study DS and DS-AD.

## Potential applications

### Mouse – iPSC chimera

Mouse – iPSC chimeric models have been used to study both DS and AD fundamental mechanisms. This approach permits the long-term growth of human cells and favours the development of complex synaptic architecture. Moreover, this combinatorial system negates the limitation of non-physiological oxygen concentrations in *in vitro* cellular systems while permitting the modelling of human-specific biology. Typically, iPSC-derived precursor cells are injected into the brain of recipient animals, but recently a more mature cell population isolated from organoids has been used [208]. In some systems *Rag2^−/−^* and/or *Il2rγ*^−/−^ mice are used to facilitate long-term maintenance of engraftment of cells by suppression of the recipient’s natural immune response to the introduced human cells, a technique first developed for hematopoietic system chimeras [276].

This chimeric approach has been used to demonstrate trisomy 21-specific changes in dendritic stability and neuronal activity [211]. Human neuronal engraftment was also used to study the role of the Hsa21 gene *OLIG2* in trisomy 21-associated learning and memory deficits via the gene’s role in GABAergic neuronal development, as had been previously reported in mice [208,277]. In AD research, a similar approach was used to understand how human neurons respond to the accumulation of Aβ [278]. In more recent years these techniques have been developed to permit the engraftment of other cell types, most notably microglia, addressing limitations of current mouse models to recapitulate key features of AD neuroinflammation. Successful long-term engraftment of this cell type, necessary to understand ageing effects, requires that the recipient mouse is both immunocompromised (*Rag2*^−/−^*Il2rγ*^−/−^) and also expresses human CSF1 (macrophage differentiation cytokine) [279]. This approach has been used to identify species-specific differences in the response of microglia to Aβ and further elucidate the role of the AD-risk gene *TREM2* [279].

Notably, these model systems are highly complex and the proliferation, survival, and differentiation of human cells after injection can vary significantly between studies with each human graft containing a different mixture of cell types [280,281]. Moreover, typically in these systems, the mouse cells are not fully replaced by the engrafted human cells which only compose a small fraction of the total brain. Mosaicism may limit the manifestation and interpretation of phenotypes in these models. Depletion of the key cell type of interest in the recipient animal could be used to mitigate this limitation. For example, diphtheria toxin receptor (DTR) expression in the lineage of interest could be utilized to ablate the cells and create a niche which can then be populated by engrafted iPSCs [282].

### iPSCs use in drug screening

Although numerous promising results of AD treatment have been obtained in animal models, there are very few medications available to treat patients, and those that are available have poor efficacy. For example, the efficacy of the recently FDA-approved immunotherapy drug aducanumab that targets Aβ is questionable [283,284]. Progress using animal model-driven drug screening approaches is very slow, with large failure rates, reflecting the limitations of these models. Primary human cells can therefore be an attractive option for drug screening [285,286]. However, due to the post-mitotic nature of many types of primary cells such as neurons and invasive procedures of cell extraction, accessing and obtaining enough primary cells can be challenging [287]. Cells differentiated from iPSCs derived from patients are a useful model for drug screening because of the patient-specific genetic background, ability to engineer isogenic controls, and ability to produce large numbers of cells [287]. Using cortical neurons differentiated from AD patient iPSCs, Kondo et al. conducted an anti-Aβ drug screen and identified a combination of compounds that may be useful for treating the earliest stages of AD [288]. More recently, through deleting one copy of Hsa21 gene *BACE2* by CRISPR-Cas9 in AD pathology-free cerebral organoids differentiated from human trisomy 21 iPSCs, Alić et al. reported an induction of AD pathology, demonstrating that *BACE2* has a protective role against AD, which could be a therapeutic target [55]. These findings also indicate that DS organoids can be a useful tool for hypothesis-free drug screening [55].

Although the use of iPSCs in drug screening has begun to identify potential drugs targeting AD, limitations of this model should not be ignored. For instance, since iPSCs are reprogrammed cells, they are epigenetically and phenotypically young and unable to well model all aspects of age-related neurodegenerative diseases, such as LOAD [287,289]. Moreover, maintenance and differentiation of iPSCs as well as validating cells differentiated from iPSCs is costly and requires a significant amount of effort [290]. Lastly, culture conditions and passage number can significantly affect phenotype, data consistency, and reproducibility [291].

### Stem cell therapies

With advancements in stem cell culture, human stem cells have become a focus of potential transplantation therapies for neurological disorders [292]. NSCs from foetal tissue transplanted into an AD mouse model reduced amyloid plaques via recruitment of activated microglia and improved performance on hippocampus-related memory tasks [293]. hESCs differentiated into BFCNs have been shown to ameliorate memory and learning deficits when transplanted into AD mouse models, showing that this subset of neurons plays a critical role and could be the target of potential therapeutics for neurological disorders, including DS and AD [294–296].

While transplants have been successful in mouse models and have provided insight into disease mechanisms, there is no evidence that current transplants are beneficial in humans. Lacking online regulation, clinics are marketing stem cell therapies with remarkable outcomes that lack results and evidence from well-controlled trials [297]. Recently, a clinic in India claimed to have successfully used stem cell transplants to treat DS in up to 14 individuals [298]. However, it is currently unknown if or how stem cells can be used to treat the genetic disorder, making it unlikely that this treatment will be beneficial but will likely put these individuals at risk of transplant-related side effects [298]. In another report, doctors injected hESCs into a child with DS, who presented with deficits in speech, motor skills and had delayed developmental milestones [299]. The report claims the child had improvements in understanding, recognition, and muscle tone and that the hESCs could have induced normal neurogenesis in the brain improving the deficits resulting from DS. However, there were no controls used in this study and no data to suggest the correction of neurogenesis [299]. Advertisements and studies claiming beneficial results of stem cell therapies can mislead individuals and their families looking for treatment options. Until we have a better understanding of the underlying mechanisms of these conditions and how to correct these alterations, cell transplants are not a beneficial treatment for DS or DS-AD in humans.

## Conclusion

Less than two decades since human iPSCs were first introduced [176–178,203], the field of disease modelling has been revolutionised and is fast developing. Compared with other preclinical models such as mouse, patient-derived iPSCs have a number of advantages for the study of human disease mechanisms. Most importantly, compared with animal studies these human-derived systems conserve fundamental human genetics and biology that may not be recapitulated in preclinical model species (such as mice and rats), thus research either *in vitro* or in combinatorial chimeric systems is likely to have high translational relevance. Moreover, iPSCs are relatively easy to obtain and have fewer ethical concerns compared with other models, such as foetal tissue, hESCs and animals [300]. Notably, *in vitro* iPSC research has considerable 3Rs (Replacement, Reduction, and Refinement) benefits and is likely to significantly reduce the number of animals used in medical research but not completely replace the need for *in vivo* research [301]. In DS-AD research, key applications include the understanding of the role of glial cells in disease pathogenesis, as key aspects of both astrocytes and microglia biology differ between mouse and human. *In vitro* iPSC and organoid research are also important for the replacement of *in vivo* research that has a particularly high animal welfare burden, such as the study of hyperexcitability and seizures in DS-AD.

However, due to the immature nature of iPSC-derived cells, it is challenging to reflect age-dependent pathological features when modelling age-related diseases, such as AD. Additionally, the majority of AD iPSC models contain fAD causal mutations which are a relatively rare cause of the disease [302]. Moreover, although the problem of heterogeneity between disease modelling and healthy control iPSCs has been largely addressed by generating isogenic controls through genome editing such as by the use of CRISPR-Cas9 technology, off-target effects of gene editing and the key role of epigenetic variations should not be ignored [303]. Despite the considerable achievements in DS-AD modelling using iPSCs, this new model of disease is still in its early stages and will have numerous obstacles to overcome. In the foreseeable future, exploring mechanisms of DS-AD will be dependent on both animal and cell models. Nevertheless, with the continuous development of techniques such as genome editing, mouse-iPSC chimeras, 3D cell culture, and multiomics, iPSC-based studies will shed more light on discovering the pathomechanisms of DS-AD and provide an efficient and reliable platform for translational medicine.

## Funding

This work was supported by the U.K. Dementia Research Institute, which receives its funding from DRI Ltd, funded by the U.K. Medical Research Council, Alzheimer’s Society and Alzheimer’s Research U.K. [grant number UKDRI-1014 (to F.K.W)]; the Alzheimer’s Research U.K. Senior Research Fellowship [grant number ARUK-SRF2018A-001 (to F.K.W)]; the Royal Society Research Grant [grant number RGS\R1\211219 (to F.K.W)]; the Wisconsin Partnership Program New Investigator Program, University of Wisconsin Alzheimer’s Disease Research Center REC Scholar Award (to A.B.); the Jerome LeJeune Foundation [grant number #1910 (to A.B.)]; the National Institute of Child Health and Human Development [grant numbers 1R01HD106197, U54 HD090256, P50HD105353 (to A.B.)]; and the Wisconsin Alumni Research Foundation (to A.B.).

## Open Access

Open access for this article was enabled by the participation of MRC Laboratory of Molecular Biology in an all-inclusive *Read & Publish* agreement with Portland Press and the Biochemical Society under a transformative agreement with *JISC*.

## Abbreviations

AD: Alzheimer’s disease
*ADAMTS1*: ADAM metallopeptidase with thrombospondin type 1 motif 1
APOE: apolipoprotein E
APP: amyloid precursor protein
Aβ: amyloid-β
BACE2: β-Secretase 2
BBB: blood–brain barrier
BFCN: basal forebrain cholinergic neuron
Cas9: CRISPR-associated protein 9
CRISPR: clustered regularly interspaced short palindromic repeats
DS: Down syndrome
DS-AD: Alzheimer’s disease in Down syndrome
DYRK1A: dual-specificity tyrosine phosphorylation-regulated kinase 1A
EOAD: early-onset AD
fAD: familial Alzheimer’s disease
GSK3: glycogen synthase kinase 3
hBMEC: human brain microvascular endothelial cell
hCMEC: human cerebral microvascular endothelial cell
HEK293: human embryonic kidney cell
hESC: human embryonic stem cell
Hsa21: human chromosome 21
GABA: gamma-aminobutyric acid
iN: induced neuron
iPSC: induced pluripotent stem cell
LOAD: late-onset AD
MAPT: microtubule-associated protein tau
NFT: neurofibrillary tangle
NGF: nerve growth factor
Mmu: Mus musculus chromosome
NPC: neural progenitor cell
NSC: neural stem cell
PSC: pluripotent stem cell
*PSEN1/2*: presenilin 1/2
sAD: sporadic AD
Trisomy 21: full or partial copy of chromosome 21
